# JAK2/STAT3/HMGCS2 signaling aggravates mitochondrial dysfunction and oxidative stress in hyperuricemia-induced cardiac dysfunction

**DOI:** 10.1186/s10020-025-01246-x

**Published:** 2025-05-13

**Authors:** Dewei Peng, Xiaoli He, Bowen Ren, Qian Wang, Lulu Peng, Yue Jiang, Shengqi Huo, Lintong Men, Wei Shi, Pengcheng Luo, Mengyin Zhu, Cuntai Zhang, Jiagao Lv, Li Lin, Sheng Li

**Affiliations:** 1 Division of Cardiology, Department of Internal Medicine, Tongji Hospital, Tongji Medical College, Huazhong University of Science and Technology, Wuhan, 430030 China; 2 Key Laboratory of Vascular Aging, Ministry of Education, Tongji Hospital, Tongji Medical College, Huazhong University of Science and Technology, 1095 Jiefang Avenue, Wuhan, 430030 P. R. China; 3 Department of Geriatrics, Tongji Hospital, Tongji Medical College, Huazhong University of Science and Technology, Wuhan, 430030 China; 4 Department of Cardiology, Central Hospital of Wuhan, Tongji Medical College, Huazhong University of Science and Technology, Wuhan, China

**Keywords:** Hyperuricemia, Cardiac dysfunction, Mitochondrial function, HMGCS2, JAK2, STAT3

## Abstract

**Background:**

High uric acid levels play a critical role in cardiovascular disease pathophysiology, being closely linked to their occurrence, progression, and prognosis. To enhance prevention and treatment of hyperuricemia-related cardiovascular diseases, understanding underlying mechanisms and identifying novel therapeutic targets are essential.

**Methods:**

A hyperuricemic mouse model was established, and transcriptomic analysis of myocardial tissue was conducted using RNA sequencing. The role of HMGCS2 in hyperuricemia-induced cardiomyocytes was investigated through HMGCS2 knockout. The transcriptional regulation of HMGCS2 by STAT3 was explored via STAT3 knockdown, overexpression, and dual-luciferase reporter assays. To further elucidate the role of the JAK2/STAT3/hmgcs2 signaling pathway in hyperuricemia-induced cardiomyocytes, we overexpressed HMGCS2 while intervening in the JAK2/STAT3 pathway in vitro. The therapeutic potential of targeting the JAK2/STAT3/HMGCS2 pathway was evaluated in hyperuricemic mice using STAT3 and JAK inhibitors to assess effects on cardiac dysfunction.

**Results:**

RNA sequencing showed significant upregulation of *HMGCS2* mRNA in hyperuricemic mouse cardiac tissue. Increased HMGCS2 protein levels were observed in cardiac tissue and AC16 cardiomyocytes. HMGCS2 knockdown alleviated uric acid-induced mitochondrial dysfunction, oxidative stress, and abnormal energy metabolism in AC16 cardiomyocytes. And high uric acid levels activate the IL-6/JAK2/STAT3 signaling pathway in AC16 cardiomyocytes, which regulates HMGCS2 expression. By modulating JAK2 and STAT3 expression and subsequently overexpressing HMGCS2, we identified the involvement of the JAK2/STAT3/HMGCS2 pathway in uric acid-induced mitochondrial dysfunction, oxidative stress, and energy metabolism abnormalities in AC16 cardiomyocytes. In vitro experiments demonstrated that intervention with the ruxolitinib and S3I-201 could ameliorate mitochondrial dysfunction, oxidative stress, and ATP levels in the heart tissue of hyperuricemic mice. Moreover, these treatments also reversed cardiac function abnormalities.

**Conclusions:**

The JAK2/STAT3/HMGCS2 pathway may contributes to uric acid-induced cardiac dysfunction by affecting mitochondrial function, oxidative stress, and ATP metabolism, offering a potential therapeutic strategy for mitigating high uric acid-induced cardiac damage.

**Graphical Abstract:**

Model of hyperuricemia-induced mitochondrial dysfunction and oxidative stress in cardiomyocytes involving JAK2/STAT3/HMGCS2 signaling.

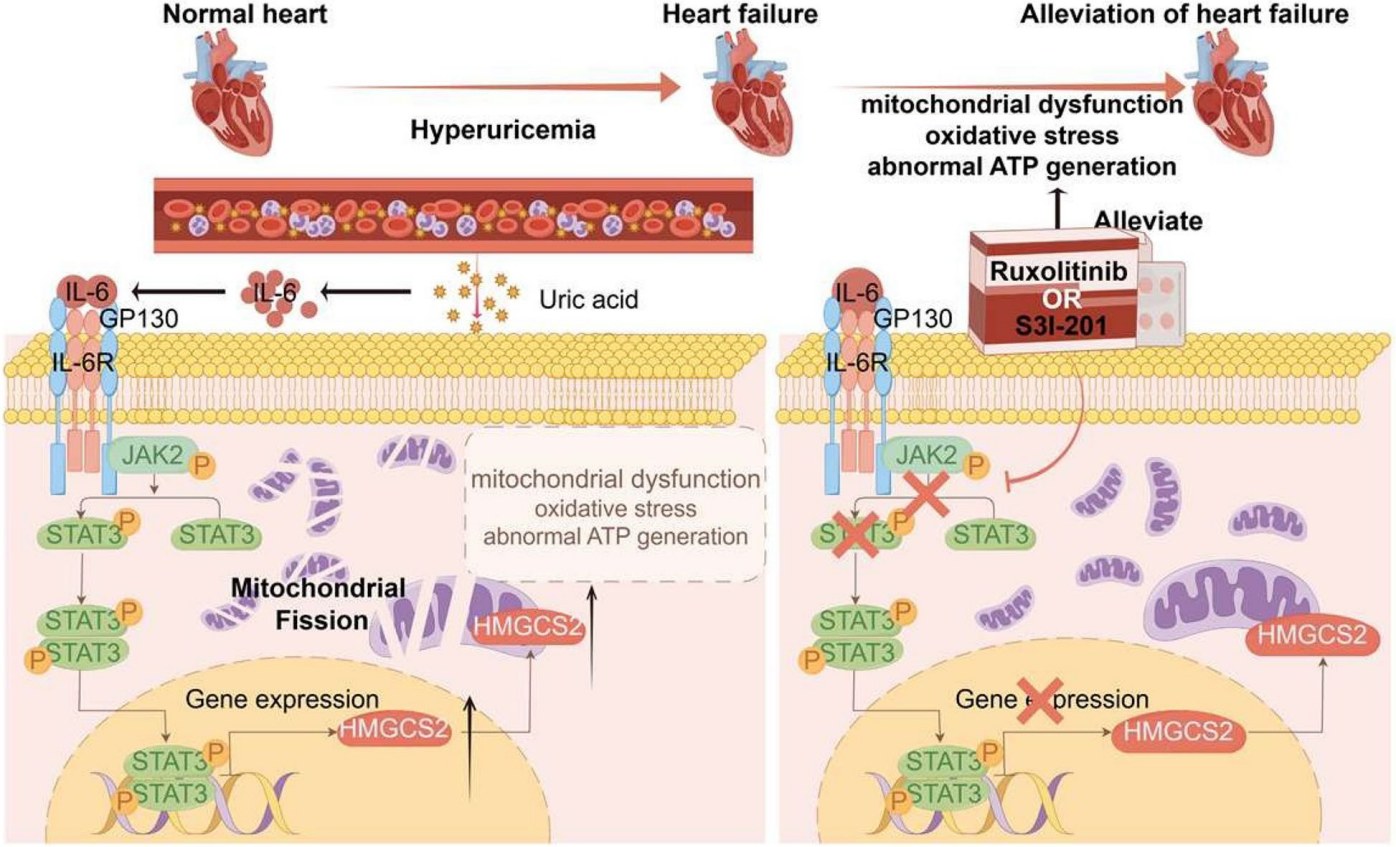

**Supplementary Information:**

The online version contains supplementary material available at 10.1186/s10020-025-01246-x.

## Highlights

1. HMGCS2 is significantly upregulated in cardiomyocytes induced by high uric acid, playing a crucial role in mitochondrial dysfunction, oxidative stress, and ATP reduction.

2. HMGCS2 induced by IL-6/JAK2/STAT3 signaling involved in mitochondrial dysfunction and oxidative stress in cardiomyocytes induced by high uric acid.

3. High uric acid activate STAT3 phosphorylation, subsequently regulating the transcription of HMGCS2.

4. Inhibiting JAK2/STAT3/HMGCS2 signaling with ruxolitinib and S3I-201 reverses cardiac dysfunction in hyperuricemic mice.

**Supplementary Information:**

The online version contains supplementary material available at 10.1186/s10020-025-01246-x.

## Introduction

Recent epidemiological studies suggest that hyperuricemia may be involved in the development of hypertension, diabetes, atherosclerosis, chronic kidney disease, atrial fibrillation (AF), and cardiovascular events (Singh et al. 2019; Kuwabara et al. 2017a; Kuwabara et al. 2017b; Kuwabara et al. 2018a; Kuwabara et al. 2018b; Maruhashi et al. 2018), and it has been identified as the fourth major cardiovascular risk factor (Virdis et al. 2020). Uric acid is closely related to the occurrence, development, and prognosis of cardiovascular diseases (Si et al. 2021). Previous studies have shown that elevated serum uric acid can promote atherosclerosis and cardiac remodeling through various complex mechanisms, including inflammation, oxidative stress, and endothelial cell damage (Kanbay et al. 2013). However, the relationship between uric acid and cardiac dysfunction remains unclear. Consequently, a comprehensive understanding of the pathogenesis of hyperuricemia-related cardiovascular diseases is imperative for the development of prevention and treatment strategies. This necessitates the identification of novel therapeutic targets and intervention methodologies.

Mitochondrial function is essential for maintaining cardiac health, as its impairment can lead to oxidative stress and energy metabolism disorders, ultimately contributing to heart dysfunction (Vagnozzi et al. 2013). Notably, mitochondrial dynamics—the balance between continuous fusion and fission—plays a critical role in preserving mitochondrial function. Disruptions in this balance, particularly excessive mitochondrial fission, are known to cause mitochondrial damage and compromise cellular health (Guo et al. 2020; Li et al. 2022a). Hyperuricemia has been shown to induce mitochondrial dysfunction and oxidative stress in cardiomyocytes, disrupting normal cellular processes (Gao et al. 2021). However, its specific role in regulating mitochondrial fission and fusion dynamics remains unclear.

To investigate the pathogenesis of hyperuricemia-related cardiovascular diseases, we performed RNA sequencing on a hyperuricemia mouse model and found that the *HMGCS2* expression level was significantly increased. 3-Hydroxy-3-methylglutaryl-CoA synthase 2 (HMGCS2) is the rate-limiting enzyme for ketone body synthesis in mitochondria (Shen et al. 2024). Ingenuity pathway analysis suggested that HMGCS2 is a central component of numerous biological networks and pathways, indicating its potential role in cardiac dysfunction associated with type 1 diabetes (Shukla et al. 2017). Studies have demonstrated that in the hearts of streptozotocin (STZ)-induced diabetic rats, HMGCS2 protein and mRNA levels are significantly elevated (Wang et al. 2020; Peng et al. 2023). The knockout of HMGCS2 has been shown to mitigate high glucose-induced myocardial cell damage by inhibiting inflammation and oxidative stress (Chen et al. 2022). Additionally, mitochondrial accumulation of HMGCS2 impairs mitochondrial function in chronic renal failure (Bai et al. 2023). Knockdown of HMGCS2 can alleviate the hyperglycemia-induced mitochondrial division of glomerular endothelial cells (Shen et al. 2024). However, whether HMGCS2 plays a significant role in uric acid-induced cardiac damage has not yet been reported.

A significant amount of evidence indicates a correlation between hyperuricemia and various inflammatory diseases (Xiao et al. 2015; Johnson et al. 2018). Clinical studies have shown that patients with hyperuricemia have significantly higher levels of inflammatory factors such as interleukin (IL-6) than do those with normal uric acid levels (Zhou et al. 2018). Elevated IL-6 level can activate the Janus kinase 2 (JAK2)/signal transducer and activator of transcription 3 (STAT3) signaling pathway, enabling STAT3 to exert its transcriptional function (Wolf et al. 2014). Recent research has demonstrated that hyperuricemia can activate the JAK2/STAT3 signaling pathway in the kidney, thereby contributing to the pathogenesis and progression of hyperuricemia-induced nephropathy (Sun et al. 2023). Several studies have demonstrated that the JAK2/STAT3 signaling pathway is involved in the regulation of mitochondrial function and oxidative stress in the heart (Li et al. 2022b; Qaed et al. 2019; Jiang et al. 2021). However, whether hyperuricemia activates the JAK2/STAT3 signaling pathway in cardiomyocytes remains to be further investigated.

This study aimed primarily to elucidate the role of HMGCS2 and the JAK2/STAT3 signaling pathway in hyperuricemia-induced mitochondrial dysfunction. Additionally, we explored whether uric acid regulates the expression of HMGCS2 through activation of the JAK2/STAT3 signaling pathway. Furthermore, this research evaluated the potential of pharmacological interventions targeting this regulatory mechanism to mitigate cardiac damage associated with elevated uric acid levels.

## Materials and methods

### Animals and reagents

All animal experiments were approved by the Animal Care and Use Committee of Tongji Hospital, Tongji Medical College, Huazhong University of Science and Technology (Approval code: TJH-202205004). Eight-week-old male wild-type C57BL/6 J mice (about 22 g) were purchased from Shulaibao Biotech. After a one-week adaptation period, the mice were randomly assigned to five groups (*n* = 5): control, hyperuricemia, hyperuricemia + allopurinol (10 mg/kg/day), hyperuricemia + ruxolitinib (5 mg/kg/day), and hyperuricemia + S3I-201 (5 mg/kg/day). A hyperuricemia mouse model was established as per the literature (Wang et al. 2023). Two weeks after induction, mice in the hyperuricemia + allopurinol group received 10 mg/kg allopurinol (HY-B0219, MedChemExpress) dissolved in 0.5% CMC-Na by gavage for four weeks. Mice in the hyperuricemia + ruxolitinib group received 5 mg/kg ruxolitinib (HY-50856, MedChemExpress) by gavage for four weeks. Mice in the hyperuricemia + S3I-201 group were treated with S3I-201 (HY-15146, MedChemExpress). The hyperuricemia and control groups received equal amounts of 0.5% CMC-Na. After six weeks, all animals were euthanized, and cardiac tissues were collected for further analysis. Serum uric acid levels were measured at the first and sixth weeks using a uric acid test kit (C012, Nanjing Jiancheng Bioengineering Institute).

### Treadmill fatigue test

Treadmill fatigue tests were performed in the sixth week. The mice were trained for three days (20 min/day) on the treadmill (ZS-PT-III, Zhongshi Technology, Shenzhen, China) with a maximum incline of 20 degrees. Initially, the treadmill speed was set at 5 m/min for 4 min, then increased to 14 m/min for 2 min. Afterward, the speed was increased by 2 m/min every minute until exhaustion. Exhaustion was defined as the mouse staying in the fatigued zone for 10 consecutive seconds despite mild electrical prodding (0.6 mA), which stopped when the mouse became exhausted. Running time and distance were recorded.

### Echocardiography

Echocardiography was performed in the sixth week using a VINNO6 high-resolution imaging system (VINNO Corporation, Suzhou, China). Mice were anesthetized with isoflurane (3% for induction, 1–2% for maintenance) and placed in a supine position with shaved chests. Left ventricular structure and function were assessed using M-mode from the short-axis view at the papillary muscle level. The measured parameters included left ventricular ejection fraction (LVEF), fractional shortening (LVFS), internal dimensions at end systole (LVIDs) and diastole (LVIDd), and left ventricular volumes at end systole (LVESV) and diastole (LVEDV). The echocardiography operators were blinded to the groups.

### Transmission electron microscopy

Myocardium samples (1 mm × 1 mm × 1 mm) were quickly excised from the left ventricle and fixed in 3% phosphate-buffered glutaraldehyde. After fixation, the samples were embedded, sectioned, and mounted for analysis. A Hitachi transmission electron microscope operating at 80 kV was used to observe and analyze the samples.

### Compounds

Uric acid with a purity of 99% (HPLC) was purchased from Sigma‒Aldrich (U2625, St. Louis, MO, USA). The uric acid powder was dissolved in a 1 M NaOH solution at a concentration of 45 mg/mL. Ruxolitinib was purchased from MCE (HY-50856, MedChemExpress) and dissolved in dimethyl sulfoxide (DMSO) at a concentration of 1 mM.

### Cell culture and treatment

AC-16 and HEK-293 cells were obtained from the Cell Bank of the Chinese Academy of Sciences. They were cultured in high-glucose Dulbecco’s modified Eagle’s medium (DMEM, KeyGEN BioTECH) with 10% fetal bovine serum (FBS, Gibco) and 1% penicillin/streptomycin (Sangon) at 37 °C with 5% CO2. The cells were treated with different concentrations (0, 100, 200, or 400 mg/L) of uric acid (U2625, Sigma) for 24 h to mimic a hyperuricemic environment. To study ruxolitinib’s effects, AC-16 cells were treated with ruxolitinib (0, 50, or 100 nM) and 400 mg/L uric acid for 24 h. After incubation, cells were harvested for western blot analysis. For soluble IL-6R (sIL-6R) experiments, AC-16 cells were treated with 400 mg/L uric acid and sIL-6R (0, 5, or 50 ng/ml) for 24 h.

### Western blot

Heart tissues and treated AC-16 cells were lysed in RIPA buffer with 1 mM protease and phosphatase inhibitors. After centrifugation at 12,000 rpm for 20 min at 4 °C, the supernatant was collected. Protein concentrations were measured using a BCA protein assay. Equal amounts of protein were separated by 10–12% SDS-PAGE and transferred to PVDF membranes. After blocking with 5% skim milk for 1 h, membranes were incubated with primary antibodies against HMGCS2 (A19232, ABclonal, 1:1000), FIS1 (10956, Proteintech, 1:1000), MFN1 (A9880, ABclonal, 1:1000), MFN2 (A19678, ABclonal, 1:1000), GAPDH (10494, Proteintech, 1:1000), P-STAT3 (9145, Cell Signaling Technology, 1:1000), T-STAT3 (12640, Cell Signaling Technology, 1:1000), P-JAK2 (68790, Cell Signaling Technology, 1:1000), and T-JAK2 (3230, Cell Signaling Technology, 1:1000). After washing, the blots were incubated with a secondary antibody (HRP-conjugated goat anti-rabbit IgG, HA1005, Promoter, 1:5000) for 1 h at room temperature. Protein detection was performed using an ECL kit (HY-K1005; MedChemExpress) on a ChemiDoc-It 510 Imager with VisionWorks software (Ultraviolet Products Ltd., Cambridge, UK).

### RNA extraction and quantitative qRT‒PCR (qPCR) analysis

The quality and concentration of RNA were measured via a spectrophotometer (NanoDrop 2000 spectrophotometer, Thermo Scientific, Waltham, MA, USA). cDNA synthesis was carried out via the use of PrimeScript™ RT Master Mix (Takara, Kusatsu, Japan). The levels of target genes were quantified via real-time fluorescence PCR with SYBR™ Select Master Mix (Thermo Fisher). The quantification of IL-6, IL-6R, and GP130 in AC-16 cells was normalized to that of GAPDH via the ΔΔCT method. The primers used were human GAPDH forward GGAGCGAGATCCCTCCAAAAT and reverse GGCTGTTGTCATACTTCTCATGG. IL-6 forward TGGTGTTGCCTGCTGCCTTC, reverse GCTGAGATGCCGTCGAGGATG. IL-6R forward AGGTCGGTGTGAACGGATTTG, reverse TGTAGACCATGTAGTTGAGGTCA. GP130 forward AGGTCGGTGTGAACGGATTTG, reverse TGTAGACCATGTAGTTGAGGTCA. The expression levels of the target genes are presented as relative fold changes compared with those in the control group.

### Transfection

For siRNA transfection, AC-16 cells were seeded in 6-well plates for 12 h and transfected with control siRNA, HMGCS2 siRNA (Robbio Co., Ltd., Guangzhou, China), or STAT3 siRNA (Robbio Co., Ltd., Guangzhou, China) using Lipo2000 (11668019; Invitrogen, U.S.). After 48 h, the cells were treated with uric acid for 24 h.

For plasmid transfection, AC-16 cells were seeded in 6-well plates for 12 h and then transfected with plasmids using Lipo3000 (L3000015; Invitrogen, U.S.). The cells were collected for Western blot analysis. The plasmids used included a control plasmid, STAT3-WT plasmid, STAT3-Y705D mutant, and STAT3-Y705 F mutant (Genechem Co., Ltd. Shanghai, China).

For lentivirus transfection, HMGCS2-overexpressing lentiviral constructs were obtained from Genomeditech (Genomeditech Co., Ltd., Shanghai, China). AC-16 cells were cultured overnight in 6-well plates and transfected with lentivirus using the transfection P reagent. After 36 h, RNA and protein were extracted, and transfected cells were selected with puromycin.

### Luciferase assays

The JASPAR database (http://jaspar.genereg.net/) was used to predict STAT3 binding sites on the HMGCS2 promoter. The wild-type and mutant HMGCS2 promoters were cloned into the pGL3-basic luciferase reporter vector (Genomeditech Co., Ltd., Shanghai, China). These constructs were cotransfected with the pRL-TK plasmid expressing Renilla luciferase (Promega Corporation, U.S.) and a STAT3 overexpression plasmid into HKE-293 cells. After 48 h, firefly and Renilla luciferase activities were measured using a Dual Luciferase Reporter Gene Assay Kit (11402ES60; Yeasen Biotech).

### Mito-tracker

AC-16 cells were treated with MitoTracker Red CMXRos (C1035; Beyotime Biotechnology) for 30 min at 37 °C. After that, the cells were rinsed with warm Hanks'balanced salt solution (HBSS, C0219; Beyotime Biotechnology). The cells were then fixed with 4% paraformaldehyde for 2 min at 37 °C. The mitochondria were visualized via a confocal microscope (Nikon, Tokyo, Japan) with a 100 × oil immersion lens.

### Measurement of the mitochondrial membrane potential (Δψm)

For cellular analysis, mitochondrial membrane potential (Δψm) was measured using the fluorescent dye JC-1 (PJC-110; Promotor Biological). After treatment, AC-16 cells were incubated with JC-1 for 30 min at 37 °C, washed with PBS, and covered with culture medium. The fluorescence signals of the JC-1 aggregates (red, 525/590 nm) and JC-1 monomers (green, 485/530 nm) were observed using an MShot fluorescence microscope (Wuhan, China). The mitochondrial membrane potential (Δψm) was quantified by the ratio of JC-1 aggregate to JC-1 monomer fluorescence.

For myocardial tissue, mitochondrial membrane potential (Δψm) was measured using Oxygraph-2 k units (Oroboros Instruments, Innsbruck). Δψm changes across various respiratory states were assessed with slight modifications to existing methods (Spinazzi et al. 2019). Briefly, Δψm was evaluated by monitoring safranin O fluorescence (5 μM) in MiR05 medium. Complex I substrates (10 mM glutamate, 2 mM malate, 2 mM pyruvate) were added, followed by 2.5 mM ADP. An inverse relationship between mitochondrial membrane potential and safranin O fluorescence was observed, with fluorescence quenched in hyperpolarized mitochondria. The maximal membrane potential was determined by measuring the difference in safranin fluorescence before and after ADP addition. The initial fluorescence, without ADP, represented the leak state. Each experiment included a calibration step with a standard curve using safranin concentrations, with maximal basal fluorescence normalized to a value of 1.

### Oxidative stress detection

ROS production was assessed using DHE (HY-D0079, MedChemExpress) staining. Fresh frozen heart sections or H9 C2 cells were stained with DHE or DCFH-DA in the dark at 37 °C for 30 min and analyzed using a MShot fluorescence microscope.

Cardiac tissues and AC-16 cells were homogenized in the respective lysis buffers from the assay kits, and the supernatants were collected for analysis. Malondialdehyde (MDA) concentration was measured using an MDA assay kit (S0131M, Beyotime Biotechnology). Superoxide dismutase (SOD) activity was evaluated using a SOD assay kit (S0101M, Beyotime Biotechnology).

### ATP assays

Intracellular ATP content was quantified via an ATP assay kit (S0026; Beyotime Biotechnology, China). The experiment was conducted according to the instructions in the kit, and luminescence was measured via a GloMax luminometer (Promega Corporation, U.S.).

### Immunohistochemistry (IHC)

After sacrifice, heart tissue was excised and fixed in 10% formalin. The fixed tissue was embedded in paraffin, sectioned, and analyzed by immunohistochemistry using IL-6-specific antibodies (ab6672, Abcam). Images were captured with an MShot microscope (Wuhan, China).

### RNA sequence

After euthanasia, cardiac tissues were isolated with four biological replicates per group. RNA-seq was conducted by BGI Genomics (Shenzhen, China). Total RNA was extracted from fresh ventricular tissue using TRIzol (Invitrogen). mRNA was purified using poly-T oligo-attached magnetic beads. Sequencing libraries were prepared with the NEBNext® Ultra™ RNA Library Prep Kit for Illumina® (NEB, USA) according to the manufacturer’s instructions, adding index codes to assign sequences to samples. The indexed samples were clustered using the TruSeq PE Cluster Kit v3-cBot HS (Illumina) on a cBot Cluster Generation System. The libraries were sequenced on an Illumina HiSeq platform with 150 bp paired-end reads. The sequencing data were filtered using SOAPnuke, removing adapters, poly-N reads, and low-quality reads to obtain clean data. Reference genome and gene model annotation files were obtained from the genome website. Ericscript (v0.5.5) and rMATS (V3.2.5) were used to detect fusion genes and differential splicing genes (DSGs). Gene expression levels were calculated using RSEM (v1.3.1). Differential expression analysis was performed with DESeq2 (v1.4.5) (or DEGseq or PoissonDis) using a Q value < 0.001. Further analysis and data mining were performed on the Dr. Tom Multiomics Data Mining System (https://biosys.bgi.com).

### Nuclear fractionation extraction

Cardiac tissues were subjected to nuclear protein extraction using the Nuclear and Cytoplasmic Protein Extraction Kit (KGP150, Keygen Biotech, China), following the manufacturer's protocol.

### Statistical analysis

The data are reported as the mean ± standard error of the mean (SEM) from a minimum of three independent experiments. Statistical analysis was conducted via GraphPad 8.0 software. Significance between groups was assessed via either one-way analysis of variance (ANOVA) or area under the curve (AUC) analysis for comparisons involving more than two groups or t tests for comparisons involving only two groups. Statistical significance was defined as a P value of less than 0.05.

## Results

### Uric acid increases mitochondrial dysfunction, oxidative stress and hmgcs2 expression in cardiomyocytes

**Fig. 1:**
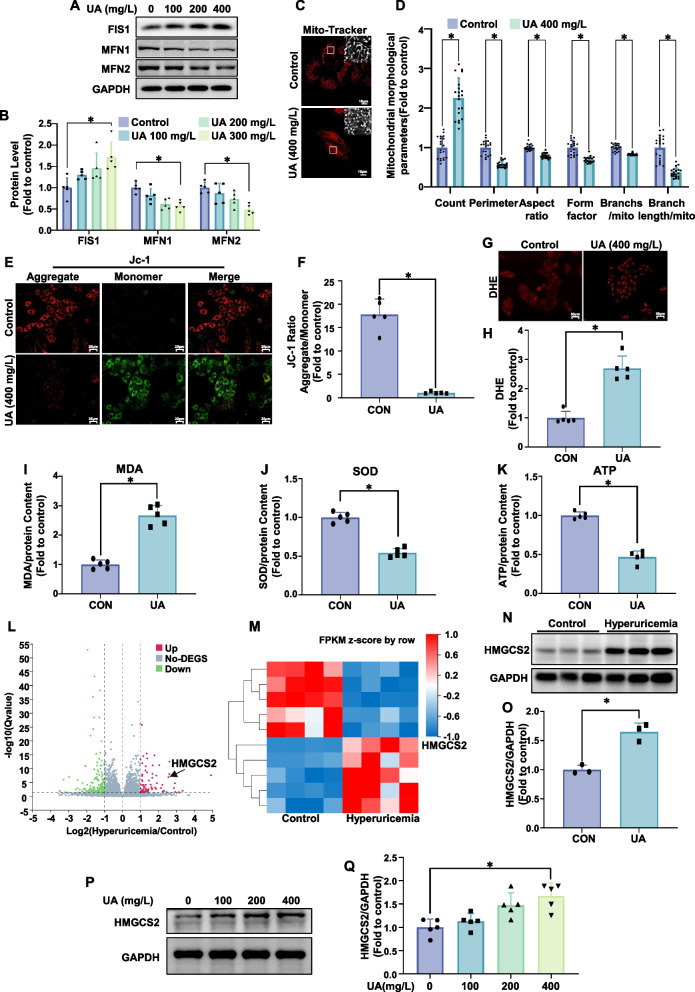
Hyperuricemia induces increased mitochondrial fission and dysfunction, elevated oxidative stress, and decreased ATP production in AC16 cardiomyocytes and increased HMGCS2 expression in cardiac tissues and cardiomyocytes. **A**, **B** Western blot analysis of FIS1, MFN1, MFN2 (**A**) protein levels in AC16 cells after uric acid treatment (0, 100, 200, 400 mg/L, 24 h). Statistical analysis (**B**) is shown (*n* = 5/group). **C**, **D **Mitochondrial morphology in AC16 cells treated with uric acid (400 mg/L, 24 h) was assessed using MitoTracker Red. Representative images (**C**, scale bar: 10 µm) and ImageJ quantification (**D**) of the number of mitochondria (count), the mean perimeter (perimeter), the mean aspect ratio (aspect ratio), the mean form factor (form factor), the number of mitochondria branches/mitochondria (Branches/mito) and the branches length/mitochondria (Branch length/mito) are shown. **E**, **F** Mitochondrial membrane potential was analyzed using JC-1 staining. Representative fluorescence images of JC-1 aggregates (red) and monomers (green) are presented (**E**, scale bar: 25 µm), along with quantification of the JC-1 aggregate/monomer ratio (**F**, *n* = 5/group). **G**, **H **Oxidative stress was detected in AC16 cells using DHE staining after uric acid treatment (400 mg/L, 24 h). Representative fluorescence images (**G**) and quantification (**H**, *n* = 5/group) are provided. **I**-**K** Measurements in AC16 cells include relative MDA content (I), SOD activity (**J**), and ATP levels (**K**) (*n* = 5/group). **L**, **M**. Volcano plot (**L**) and heatmap (**M**) of differentially expressed genes in myocardial tissue from hyperuricemic mice versus controls (*n* = 4 mice/group). **N**, **O **Representative western blot image (**N**) and corresponding statistical analysis (*n* = 3 mice/group) (**O**) of HMGCS2 protein levels in the myocardial tissue of hyperuricemic model mice. P-Q. Western blot analysis of HMGCS2 (**P**) protein levels in AC16 cells after uric acid treatment (0, 100, 200, 400 mg/L, 24 h). Statistical analysis (**Q**) is shown (*n* = 5/group)

To investigate the mechanisms of hyperuricemia-related cardiovascular diseases, we examined the expression of mitochondrial fission 1 protein (FIS1), as well as mitofusin 1 (MFN1) and mitofusin 2 (MFN2), which are key regulators of mitochondrial fusion. We found that the expression level of FIS1 was increased, while the expression levels of MFN1 and MFN2 were markedly decreased in AC-16 cells treated with 400 mg/L uric acid (Fig. 1A, B). Mitochondrial fluorescence staining revealed notable morphological changes in mitochondria upon uric acid treatment compared to controls (Fig. 1C). ImageJ analysis of mitochondrial morphology (Fig. 1D) showed an increase in mitochondrial count, along with reduced average mitochondrial perimeter, aspect ratio, and form factor. The total branch length per mitochondrion was also significantly reduced, confirming that uric acid induces mitochondrial fragmentation.

We further assessed mitochondrial membrane potential, finding that uric acid significantly decreased it in AC-16 cells compared to controls (Fig. 1E, F). Oxidative stress levels were evaluated in uric acid-treated AC-16 cells. Dihydroethidium (DHE) staining (Fig. 1G, H) and malondialdehyde (MDA) assays (Fig. 1I) revealed elevated oxidative stress and lipid peroxidation, while superoxide dismutase (SOD) activity assays (Fig. 1J) showed significantly reduced SOD activity. In uric acid-treated AC-16 cells, mitochondrial fission was enhanced, mitochondrial dysfunction was observed, and oxidative stress levels were elevated. ATP levels were also measured, showing a significant reduction in uric acid-treated cells compared to controls (Fig. 1K).

To explore the underlying mechanisms, we established a hyperuricemic mouse model and performed RNA sequencing on heart tissues. RNA sequencing (Fig. 1L, M) identified the top ten differentially expressed genes (DEGs) with |log2 FC|> 1 and a Q value < 0.001, among which *HMGCS2* was selected for further analysis. Western blot confirmed significantly elevated HMGCS2 protein levels in hyperuricemic mouse myocardial tissues compared to controls (Fig. 1N, O). In vitro, treatment of AC-16 cardiomyocytes with uric acid for 24 h increased HMGCS2 protein expression, particularly at 400 mg/L (Fig. 1P, Q). These results indicate that hyperuricemia significantly upregulates the expression of HMGCS2 in cardiomyocytes.

### Knockdown of HMGCS2 mitigates uric acid-induced mitochondrial dysfunction and oxidative stress

**Fig. 2:**
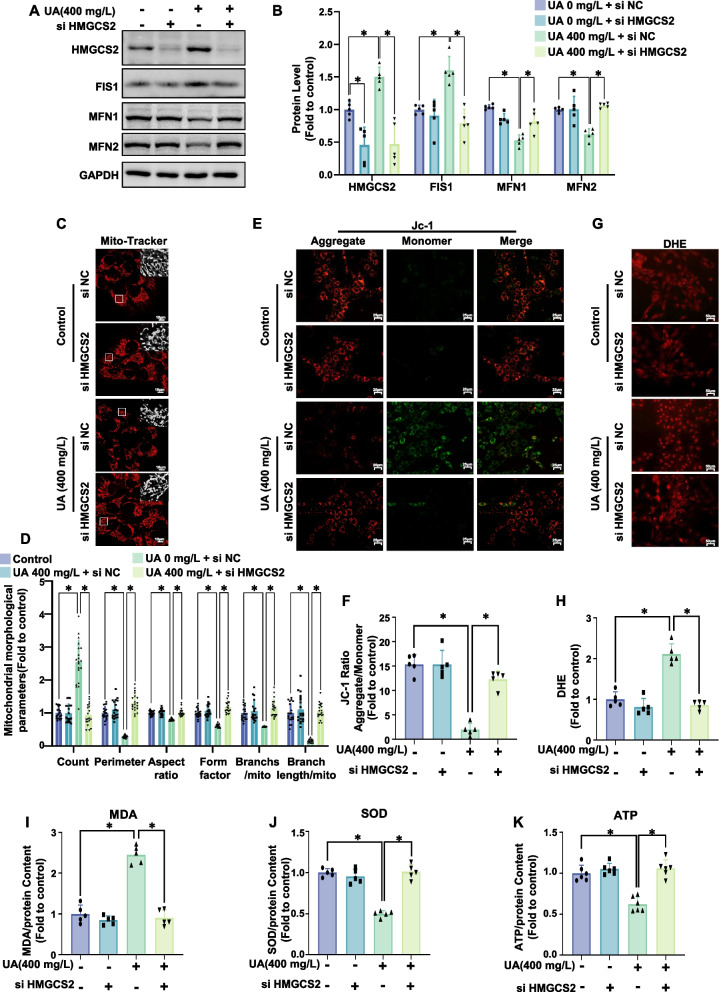
HMGCS2 knockdown prevented uric acid-induced mitochondrial fission and dysfunction and oxidative stress and alleviated ATP production in AC16 cardiomyocytes. AC16 cells were transfected with control siRNA or HMGCS2 siRNA (siHMGCS2) and then treated with uric acid (400 mg/L) for 24 h. **A**, **B **Representative Western blot image (**A**) and the corresponding statistical analysis (*n* = 5/group) of HMGCS2, FIS1, MFN1, MFN2 (**B**) protein levels in AC16 cells. **C**, **D **Mitochondrial morphology in AC16 cells treated with uric acid (400 mg/L, 24 h) was assessed using MitoTracker Red. Representative images (**C**, scale bar: 10 µm) and ImageJ quantification (**D**) of the number of mitochondria (count), the mean perimeter (perimeter), the mean aspect ratio (aspect ratio), the mean form factor (form factor), the number of mitochondria branches/mitochondria (Branches/mito) and the branches length/mitochondria (Branch length/mito) are shown. **E**, **F **Mitochondrial membrane potential was analyzed using JC-1 staining. Representative fluorescence images of JC-1 aggregates (red) and monomers (green) are presented (**E**, scale bar: 25 µm), along with quantification of the JC-1 aggregate/monomer ratio (**F**, *n* = 5/group). **G**, **H **DHE staining was used to detect oxidative stress in AC16 cells. Representative fluorescence images (**G**) and the corresponding statistical analysis (*n* = 5/group) (**H**) are shown in the figure. I Relative MDA content in AC16 cells (*n* = 5/group). **J** Relative level of SOD in AC16 cells (*n* = 5/group). K. Relative ATP content in AC16 cells (*n* = 5/group). The data represent the means ± S.E.M.s. *p < 0.05 vs the indicated group

To verify HMGCS2's role in uric acid-induced mitochondrial dysfunction and oxidative stress, we used HMGCS2 siRNA (siHMGCS2) to knock down HMGCS2 in AC-16 cells and examined its effects on mitochondrial fission, function, oxidative stress, and ATP production after uric acid treatment. siHMGCS2 significantly reduced uric acid-induced expression of HMGCS2 and FIS1, and restored the expression levels of MFN1 and MFN2 that were suppressed by uric acid treatment. (Fig. 2A, B). Mitochondrial staining (Fig. 2C, D) and JC-1 staining (Fig. 2E, F) showed that siHMGCS2 inhibited uric acid-induced mitochondrial fission and restored mitochondrial membrane potential. Additionally, DHE staining (Fig. 2G, H), MDA assays (Fig. 2I), and SOD activity assays (Fig. 2J) demonstrated that siHMGCS2 reduced oxidative stress and lipid peroxidation while normalizing SOD activity. ATP assays (Fig. 2K) further revealed that siHMGCS2 restored ATP levels reduced by uric acid. These results suggest that HMGCS2 may play a critical role in uric acid-induced cardiac dysfunction.

### Uric acid activates the JAK2/STAT3 pathway to regulate hmgcs2 expression

**Fig. 3:**
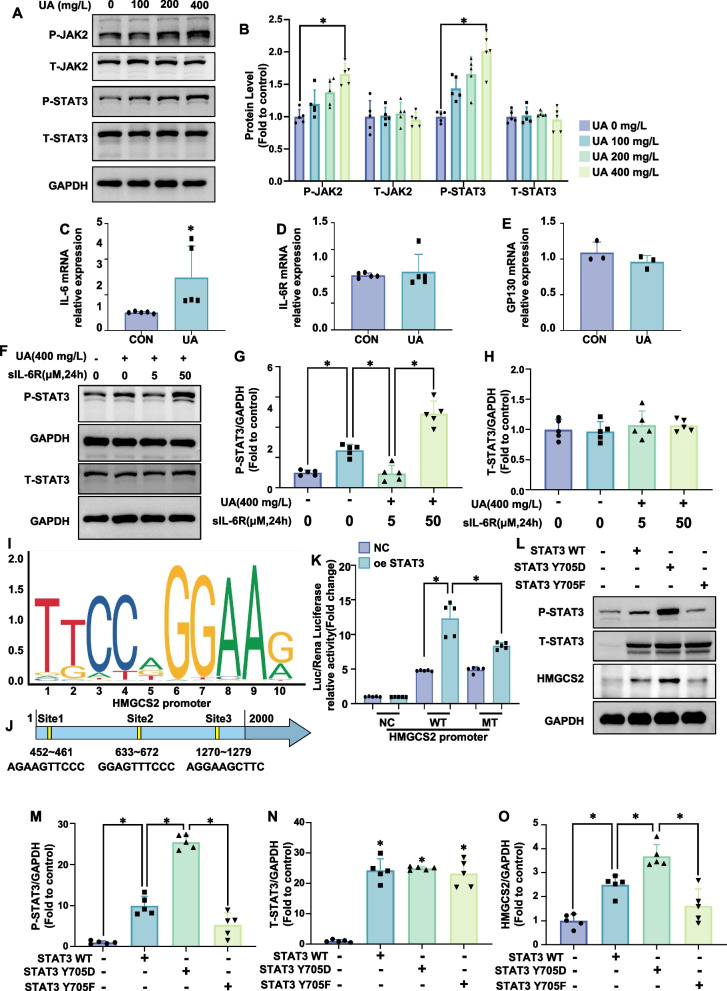
Uric acid activates the JAK2/STAT3 pathway to regulate HMGCS2 expression. **A**, **B **AC16 cells were treated with uric acid (0, 100, 200, or 400 mg/L) for 24 h. Western blotting was used to determine the levels of P-JAK2, T-JAK2, P-STAT3, and T-STAT3, and representative western blot images (**A**) and the corresponding statistical analysis (**B**) (*n* = 5/group) are presented in the figure. **C** Quantification of the IL-6 mRNA level (*n* = 5/group). **D** Quantification of the IL-6R mRNA level (*n* = 6/group). **E** Quantification of the GP130 mRNA level (*n* = 3/group). **F**–**H **The effects of various concentrations of sIL-6R on the activation of STAT3 induced by uric acid (400 mg/L). AC16 cells were cotreated with uric acid (400 mg/L) and sIL-6R (0, 5, or 50 ng/ml) for 24 h. Representative western blot images (**F**) and quantification (*n* = 5/group) of P-STAT3 (**G**) and STAT3 (**H**) are shown in the figure. **I**, **J** STAT3 motif (**I**) and STAT3-binding site (**J**) in the HMGCS2 promoter. **K** HEK-293 cells were transfected with a STAT3-overexpressing plasmid, a wild-type (WT) HMGCS2 promoter or a mutant (MT) HMGCS2 promoter plasmid for 48 h. Luciferase activity was measured via a dual-luciferase assay to detect the combined effects of STAT3 and the HMGCS2 promoter in HEK-293 cells (*n* = 5/group). **L**, **O** AC16 cells were individually transfected with the control plasmid, wild-type STAT3 plasmid (STAT3 WT), Y705D mutant STAT3 (STAT3 Y705D), or Y705 F mutant STAT3 (STAT3 Y705 F) for 48 h, and representative western blot images (**A**) and corresponding statistical analysis (**M**-**O**) (*n* = 3/group) are presented in the figure (*n* = 3/group). The data represent the means ± S.E.M.s. *p < 0.05 vs the indicated group

To investigate uric acid's regulatory role in HMGCS2 expression via JAK2/STAT3 pathway activation, we assessed JAK2 and STAT3 activation in cardiomyocytes treated with uric acid. Uric acid significantly increased JAK2-Y1007/Y1008 and STAT3-Y705 phosphorylation without altering total JAK2 and STAT3 levels (Fig. 3A, B). In AC-16 cells, immunofluorescence analysis revealed that uric acid promoted the nuclear translocation of STAT3 compared to the control group (Supplementary Fig. 1A). Furthermore, Western blot analysis demonstrated that, in the myocardial tissue of hyperuricemic mice, the nuclear expression level of STAT3 was significantly increased relative to the control group (Supplementary Fig. 1B). PCR analysis revealed elevated IL-6 mRNA levels in uric acid-treated AC-16 cells, while IL-6R and GP130 mRNA levels remained unchanged (Fig. 3C-E). IL-6 binds to soluble IL-6R (sIL-6R) with an affinity comparable to that of membrane-bound IL-6R (mIL-6R). At low concentrations, sIL-6R binds to IL-6 and exerts an antagonistic effect, while high concentrations of the IL-6/sIL-6R complex activate membrane gp130, leading to subsequent JAK/STAT3 phosphorylation (Jiang et al. 2021; Shi et al. 2020). Our study found that low sIL-6R concentrations inhibited uric acid-induced STAT3-Y705 phosphorylation in AC-16 cells, whereas high concentrations enhanced it (Fig. 3F-H).

**Fig. 4:**
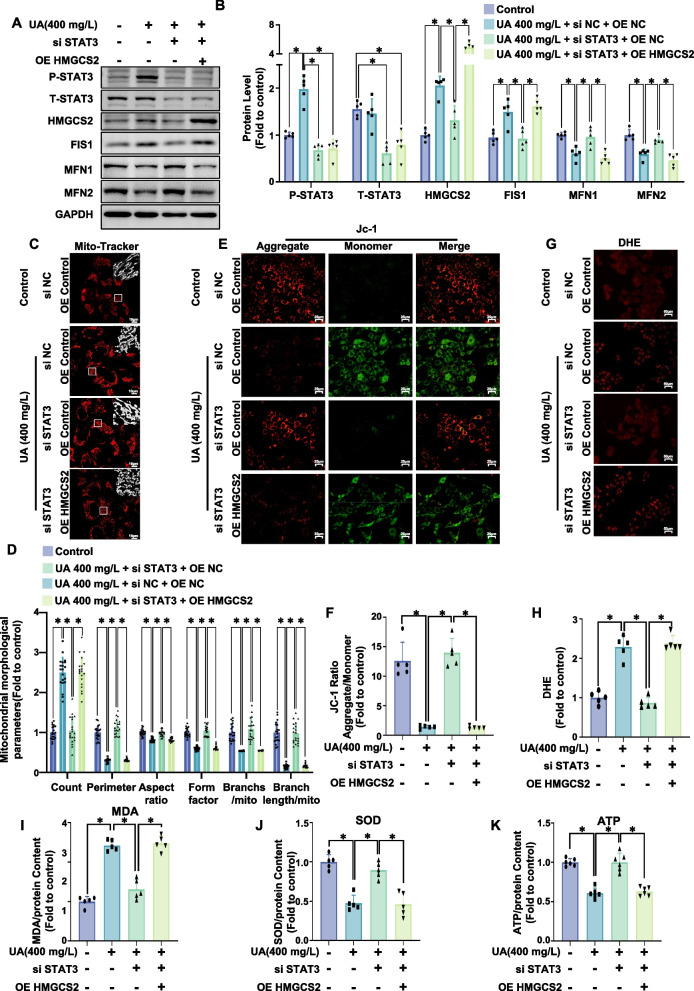
STAT3 knockdown prevented uric acid-induced mitochondrial fission and dysfunction and oxidative stress and alleviated ATP production in AC16 cardiomyocytes, and HMGCS2 overexpression reversed these effects. AC16 cells were first treated with a combination of siRNA (si NC or si STAT3) and plasmids (OE NC or OE HMGCS2) under different conditions and then treated with uric acid (400 mg/L). **A**, **B** Representative western blot image (**A**) and the corresponding statistical analysis (*n* = 5/group) (**B**) of P-STAT3, T-STAT3, HMGCS2, FIS1, MFN1, MFN2 protein levels. **C**, **D** Mitochondrial morphology in AC16 cells treated with uric acid (400 mg/L, 24 h) was assessed using MitoTracker Red. Representative images (**C**, scale bar: 10 µm) and ImageJ quantification (**D**) of the number of mitochondria (count), the mean perimeter (perimeter), the mean aspect ratio (aspect ratio), the mean form factor (form factor), the number of mitochondria branches/mitochondria (Branches/mito) and the branches length/mitochondria (Branch length/mito) are shown. **E**, **F** Mitochondrial membrane potential was analyzed using JC-1 staining. Representative fluorescence images of JC-1 aggregates (red) and monomers (green) are presented (**E**, scale bar: 25 µm), along with quantification of the JC-1 aggregate/monomer ratio (**F**, *n* = 5/group). **G**, **H **(**G**) Representative images of DHE staining. and the corresponding statistical analysis (*n* = 5/group). Scale bar: 50 µm. **I** Relative MDA content in AC16 cells (*n* = 5/group). **J** Relative SOD activity in AC16 cells (*n* = 3/group). K. Relative ATP content in AC16 cells (*n* = 5/group). The data represent the means ± S.E.M.s. *p < 0.05 vs the indicated group

To explore the effect of STAT3 on HMGCS2, we identified the STAT3 binding site on the HMGCS2 promoter using the JASPAR database. This was further validated in HEK-293 cells through a dual-luciferase reporter assay, which revealed that STAT3 overexpression increased the activity of the HMGCS2 WT promoter but not the mutant promoter (HMGCS2 MT). (Fig. 3I-K). Plasmids overexpressing STAT3-WT, STAT3-Y705D, and STAT3-Y705 F were transfected into AC-16 cells. Western blot analysis showed all plasmids increased total STAT3 expression. STAT3-Y705D significantly enhanced STAT3-Y705 phosphorylation and HMGCS2 expression, whereas STAT3-Y705 F inhibited STAT3-Y705 phosphorylation, with no significant change in HMGCS2 expression compared to controls (Fig. 3L-O). STAT3 knockdown via siSTAT3 significantly reduced HMGCS2 protein levels in uric acid-treated myocardial cells (Fig. 4A).

### The STAT3/HMGCS2 axis is critical in hyperuricemia-induced mitochondrial dysfunction and oxidative stress

To further explore the role of the STAT3/HMGCS2 axis in uric acid-induced mitochondrial dysfunction and oxidative stress, STAT3 was knocked down in AC-16 cells using siSTAT3, and HMGCS2-overexpressing AC-16 cells were constructed. Western blot analysis showed that siSTAT3 significantly reduced uric acid-induced FIS1 expression and restored the expression levels of MFN1 and MFN2 suppressed by uric acid. However, these effects were not observed in HMGCS2-overexpressing cells (Fig. 4A, B). Mito-Tracker and JC-1 staining revealed that siSTAT3 alleviated uric acid-induced mitochondrial fission and restored mitochondrial membrane potential, effects reversed by HMGCS2 overexpression (Fig. 4C-F). DHE staining, along with MDA and SOD assays, demonstrated that siSTAT3 reduced uric acid-induced oxidative stress in normal cells, but not in HMGCS2-overexpressing cells (Fig. 4G-J). Additionally, ATP measurements showed that siSTAT3 reversed the uric acid-induced reduction in ATP levels, which was counteracted by HMGCS2 overexpression (Fig. 4K). In conclusion, siSTAT3 mitigated uric acid-induced mitochondrial fission, dysfunction, oxidative stress, and ATP depletion, while HMGCS2 overexpression negated these effects. These findings point to the potential role of the STAT3/HMGCS2 axis in hyperuricemia-induced mitochondrial dysfunction and oxidative stress.

### JAK inhibitors alleviated mitochondrial dysfunction and oxidative stress in response to hyperuricemia

**Fig. 5:**
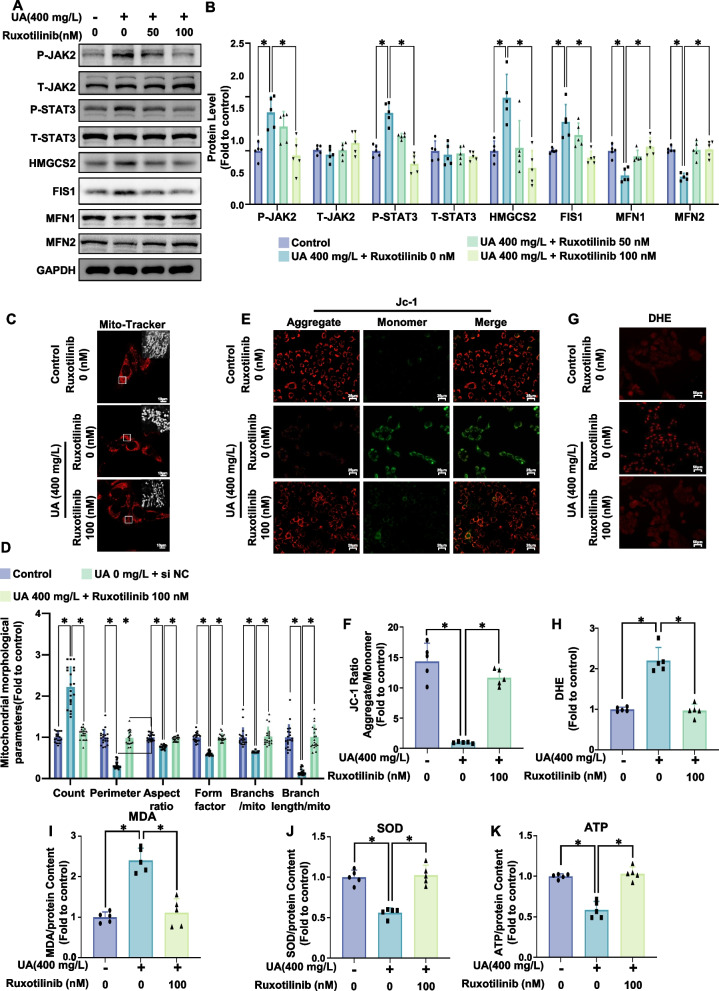
The JAK inhibitor ruxotilinib prevented uric acid-induced mitochondrial fission and dysfunction and oxidative stress and alleviated ATP production in AC16 cardiomyocytes. AC16 cells were cotreated with uric acid (400 mg/L) and ruxotilinib (0, 50, 100 nM) for 24 h. **A**, **B** Representative western blot image (**A**) and the corresponding statistical analysis (*n* = 5/group) (**B**) of P-JAK2, T-JAK2, P-STAT3, T-STAT, HMGCS2, FIS1, MFN1, MFN2 protein levels. **C**, **D** Mitochondrial morphology in AC16 cells treated with uric acid (400 mg/L, 24 h) was assessed using MitoTracker Red. Representative images (**C**, scale bar: 10 µm) and ImageJ quantification (**D**) of the number of mitochondria (count), the mean perimeter (perimeter), the mean aspect ratio (aspect ratio), the mean form factor (form factor), the number of mitochondria branches/mitochondria (Branches/mito) and the branches length/mitochondria (Branch length/mito) are shown. **E**, **F** Mitochondrial membrane potential was analyzed using JC-1 staining. Representative fluorescence images of JC-1 aggregates (red) and monomers (green) are presented (**E**, scale bar: 25 µm), along with quantification of the JC-1 aggregate/monomer ratio (**F**, *n* = 5/group). **G**, **H **(**G**) Representative images of DHE staining. and the corresponding statistical analysis (*n* = 5/group). Scale bar: 50 µm. **I** Relative MDA content in AC16 cells (*n* = 5/group). **J** Relative SOD activity in AC16 cells (*n* = 5/group). **K** Relative ATP content in AC16 cells (*n* = 5/group). The data represent the means ± S.E.M.s. *p < 0.05 vs the indicated group

**Fig. 6:**
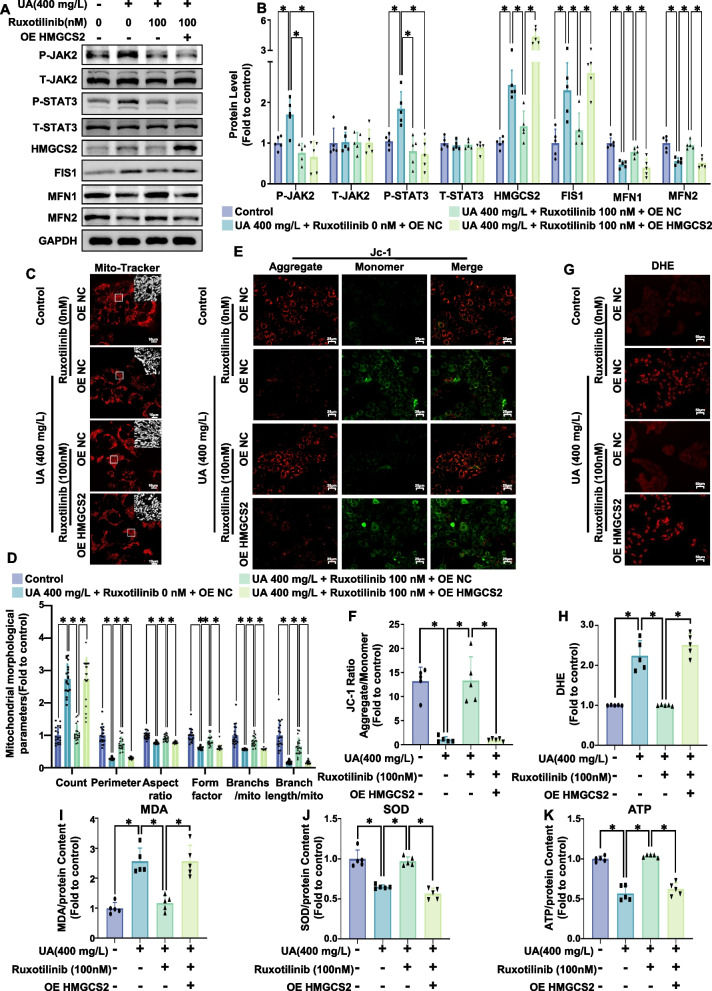
The JAK inhibitor ruxotilinib prevented uric acid-induced mitochondrial fission and dysfunction and oxidative stress and alleviated ATP production in AC16 cardiomyocytes. AC16 cells were first treated with plasmids (OE NC or OE HMGCS2) and then cotreated with uric acid (400 mg/L) and ruxotilinib (0, 50, or 100 nM) for 24 h. **A**, **B** Representative western blot image (**A**) and the corresponding statistical analysis (*n* = 5/group) (**B**) of P-JAK2, T-JAK2, P-STAT3, T-STAT, HMGCS2, FIS1, MFN1, MFN2 protein levels. **C**, **D** Mitochondrial morphology in AC16 cells treated with uric acid (400 mg/L, 24 h) was assessed using MitoTracker Red. Representative images (C, scale bar: 10 µm) and ImageJ quantification (**D**) of the number of mitochondria (count), the mean perimeter (perimeter), the mean aspect ratio (aspect ratio), the mean form factor (form factor), the number of mitochondria branches/mitochondria (Branches/mito) and the branches length/mitochondria (Branch length/mito) are shown. **E**, **F** Mitochondrial membrane potential was analyzed using JC-1 staining. Representative fluorescence images of JC-1 aggregates (red) and monomers (green) are presented (**E**, scale bar: 25 µm), along with quantification of the JC-1 aggregate/monomer ratio (**F**, *n* = 5/group). **G**, **H** Representative images of DHE-stained samples (**G**) and the corresponding statistical analysis (*n* = 5/group) are shown. Scale bar: 50 µm. **I** Relative MDA content in AC16 cells (*n* = 5/group). **J** Relative SOD activity in AC16 cells (*n* = 5/group). **K** Relative ATP content in AC16 cells (*n* = 5/group). The data represent the means ± S.E.M.s. *p < 0.05 vs the indicated group

Next, we investigated whether JAK2 plays a crucial role in hyperuricemia-induced mitochondrial dysfunction and oxidative stress. We used ruxolitinib, a JAK inhibitor widely used in clinical practice, for our experiments. Our results revealed that ruxolitinib significantly reduced uric acid-induced phosphorylation of JAK2 and STAT3 (Fig. 5A, B). Additionally, ruxolitinib reduced the protein expression levels of HMGCS2 and the mitochondrial fission-related protein FIS1, while restoring the expression levels of MFN1 and MFN2 that were suppressed by uric acid (Fig. 5A, B). Mitochondrial staining of AC-16 cells revealed that ruxolitinib significantly alleviated uric acid-induced mitochondrial fission (Fig. 5C, D). The results of JC-1 staining indicated that ruxolitinib reversed the decrease in the mitochondrial membrane potential induced by uric acid (Fig. 6E, F). In addition, DHE staining and MDA and SOD assays revealed that ruxolitinib significantly reduced uric acid-induced oxidative stress (Fig. 5G-J). Finally, ruxolitinib also significantly restored the uric acid-induced increase in ATP production in AC-16 cells (Fig. 5K). These results suggest that JAK2 may be involved in hyperuricemia-induced mitochondrial dysfunction and oxidative stress through the STAT3/HMGCS2 signaling axis.

### Overexpression of HMGCS2 abrogates the therapeutic efficacy of JAK inhibitors against hyperuricemia-induced disruption in vitro

To further investigate whether the JAK inhibitor ruxolitinib alleviates hyperuricemia-induced mitochondrial dysfunction and oxidative stress by regulating HMGCS2 expression, we conducted experiments in which ruxolitinib was used to treat uric acid-induced normal cells and HMGCS2 stably overexpressing AC-16 cells. We found that overexpressing HMGCS2 had no significant effect on the phosphorylation levels of JAK2 and STAT3 (Fig. 6A, B). However, HMGCS2 overexpression markedly abrogated the effects of ruxolitinib on uric acid-induced mitochondrial fission and improved the mitochondrial membrane potential (Fig. 6C-F). Furthermore, HMGCS2 overexpression also counteracted the therapeutic effects of ruxolitinib on uric acid-induced oxidative stress and ATP production (Fig. 6G-K). Taken together, these results suggest that JAK2 participates in uric acid-induced mitochondrial dysfunction and oxidative stress by activating the STAT3/HMGCS2 signaling axis. In addition, in vitro, the JAK inhibitor ruxolitinib can alleviate hyperuricemia-induced mitochondrial dysfunction and oxidative stress by affecting the JAK2/STAT3/HMGCS2 signaling pathway.

### JAK and STAT3 inhibitors suppressed the JAK2/STAT3/HMGCS2 signaling pathway in hyperuricemia mice

A hyperuricemic mouse model was established and treated with the JAK inhibitor ruxolitinib and the STAT3 inhibitor S3I-201 to modulate the JAK2/STAT3 signaling pathway, with allopurinol serving as a positive control. Serum uric acid levels measured one week after model induction showed a significant increase in hyperuricemic mice (Supplementary Fig. 2A). From the second week post-induction, mice were administered allopurinol (10 mg/kg), ruxolitinib (5 mg/kg), or S3I-201 (5 mg/kg) for 4 weeks. At the end of treatment, allopurinol significantly reduced serum uric acid levels, while ruxolitinib and S3I-201 had no significant effect (Supplementary Fig. 2B).

**Fig. 7:**
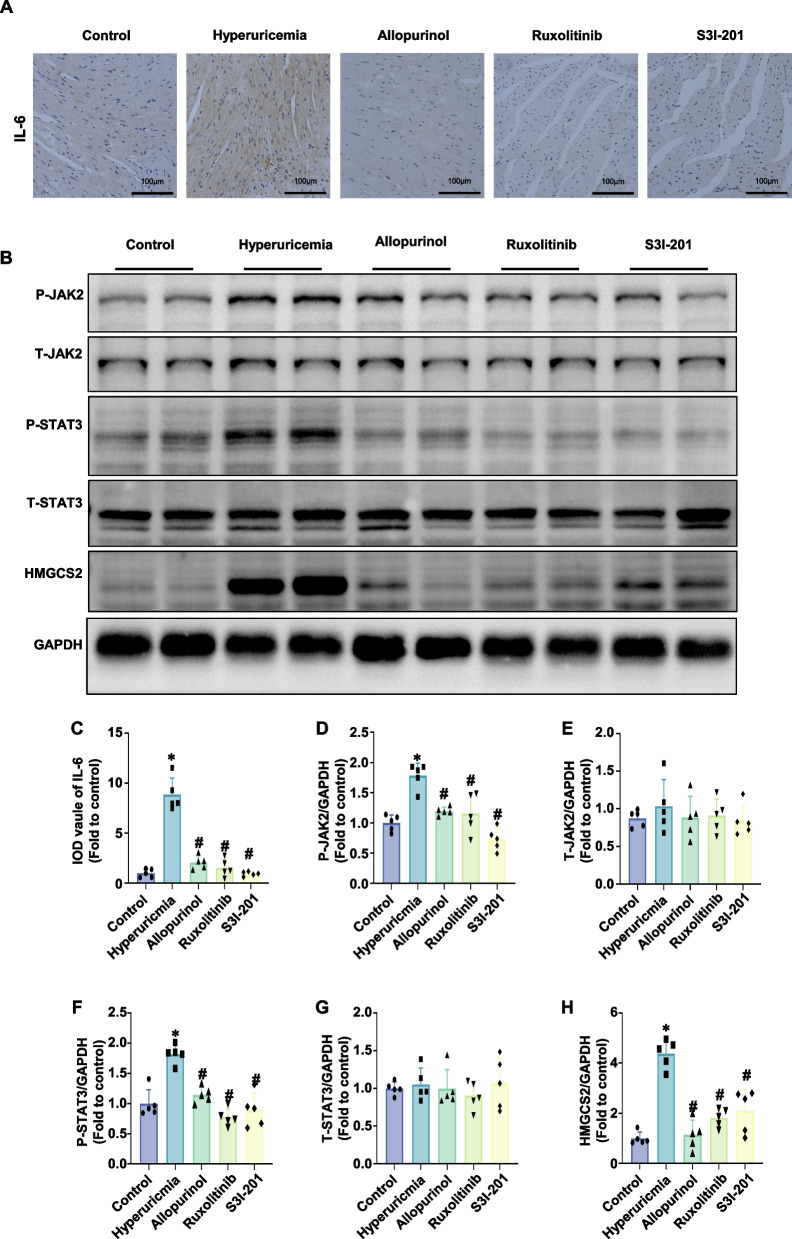
JAK and STAT3 inhibitors attenuated the JAK2/STAT3/HMGCS2 signaling pathway in the hearts of hyperuricemic mice. **A** and **C** Representative immunohistochemical images of IL-6 (brown) in mouse heart tissues (scale bar: 100 µm) (**A**) and the corresponding statistical analysis (**C**) (*n* = 5 mice/group). **B** Representative western blot images of P-JAK2, T-JAK2, P-STAT3, T-STAT, and HMGCS2 protein levels. **D**-**H** Quantitative statistical diagrams of P-JAK2 (**D**), T-JAK2 (**E**), P-STAT3 (**F**), T-STAT (**G**), and HMGCS2 (**H**) detected by western blot (*n* = 5 mice/group). The data represent the means ± S.E.M.s. *p < 0.05 vs the control group. #*p* < 0.05 vs the hyperuricemia group

Immunohistochemistry revealed significantly elevated IL-6 levels in the hearts of hyperuricemic mice compared to controls, which were reduced by allopurinol, ruxolitinib, and S3I-201 treatments (Fig. 7A, C). Western blot analysis showed that hyperuricemia significantly increased JAK2 and STAT3 phosphorylation in cardiac tissue without altering total protein levels (Fig. 7B, D-G). Allopurinol, ruxolitinib, and S3I-201 effectively reduced JAK2 and STAT3 phosphorylation (Fig. 7B, D-G) and decreased HMGCS2 protein expression in hyperuricemic mouse hearts (Fig. 7B, H).

These results indicate that hyperuricemia activates the JAK2/STAT3/HMGCS2 signaling pathway in cardiac tissue and that inhibiting JAK2 and STAT3 phosphorylation effectively suppresses this activation.

### Inhibition of the JAK2/STAT3/HMGCS2 signaling pathway mitigated mitochondrial dysfunction and oxidative stress and improved cardiac function

**Fig. 8:**
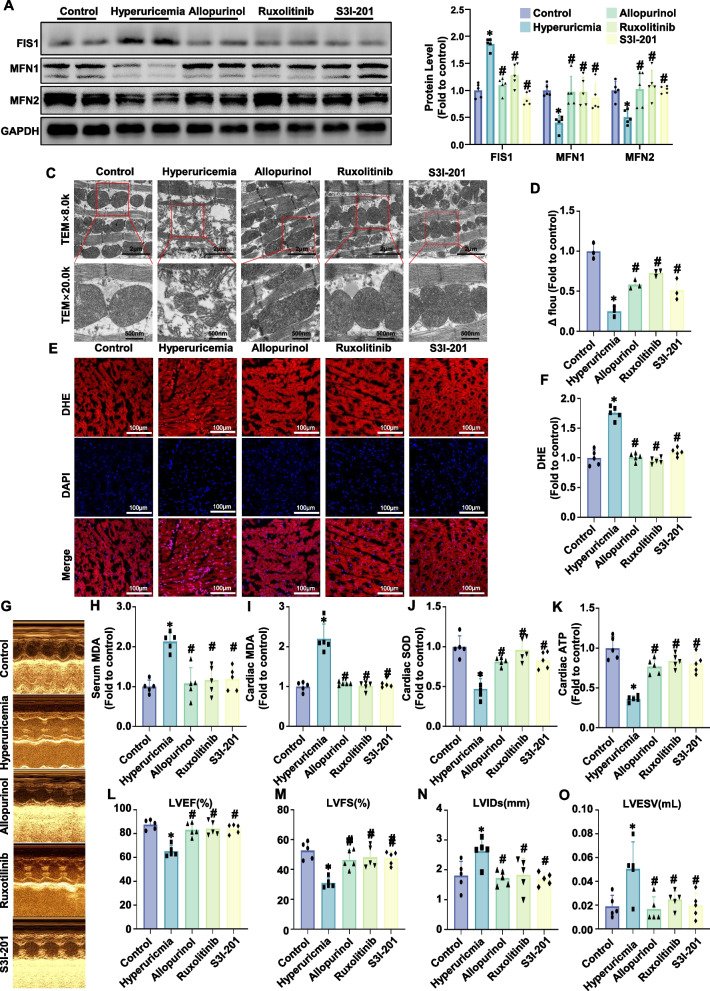
JAK and STAT3 inhibitors alleviated mitochondrial dysfunction and oxidative stress and improved cardiac function. **A**, **B** Representative western blot image (**A**) and the corresponding statistical analysis (*n* = 5 mice/group) (**B**) of FIS1, MFN1, MFN2 protein levels. **C** Representative transmission electron microscopy images of mitochondria in cardiomyocytes. Scale bar: 500 nm. **D** Mitochondrial membrane potential (Δ flou) in cardiac mitochondria determined via safranin (*n* = 3 mice/group). **E**, **F** Representative images of DHE staining (**E**) and the corresponding statistical analysis (**F**) (*n* = 5 mice/group). Scale bar: 50 µm. **G** Representative echocardiographic images from the parasternal short-axis view were used to analyze the cardiac function of the mice at the sixth week after hyperuricemic induction. **H** Relative MDA content in mouse serum (*n* = 5/group). **I** Relative MDA content in mouse hearts (*n* = 5/group). **J** Relative SOD activity in mouse hearts (*n* = 5/group). **K** Relative ATP content in mouse hearts (*n* = 5/group). **L**-**O** Representative left ventricular M-mode images. **L** Left ventricular ejection fraction (LVEF). **M** Left ventricular fractional shortening (LVFS). **N **Left ventricular internal dimension in systole (LVIDs). **O** Left ventricular internal dimension in systole (LVIDs). The data represent the means ± S.E.M.s. *p < 0.05 vs the control group. #*p* < 0.05 vs the hyperuricemia group

Finally, we evaluated whether inhibiting the JAK2/STAT3/HMGCS2 signaling pathway could mitigate mitochondrial dysfunction, oxidative stress, and cardiac dysfunction in hyperuricemic mice. Firstly, we evaluated the expression of the mitochondrial fission-related protein Fis1 and the mitochondrial fusion proteins MFN1 and MFN2 in myocardial tissue. Compared to the control group, hyperuricemic mice exhibited a significant increase in FIS1 protein expression, while the protein levels of MFN1 and MFN2 were markedly decreased. Treatment with allopurinol, ruxolitinib, or S3I-201 reduced these elevated levels (Fig. 8A, B). Electron microscopy revealed severe mitochondrial damage in cardiomyocytes of hyperuricemic mice, which was normalized by treatment with allopurinol, ruxolitinib, or S3I-201 (Fig. 8C). Mitochondrial membrane potential assays showed a significant reduction in hyperuricemic mice, which was restored by these treatments (Fig. 8D).

We then measured oxidative stress in heart tissue. DHE staining revealed higher oxidative stress levels in hyperuricemic mice compared to controls (Fig. 8E, F). Serum and cardiac MDA levels were also elevated, while SOD activity was reduced in hyperuricemic mice (Fig. 8H-J). Treatment with allopurinol, ruxolitinib, or S3I-201 reversed these oxidative stress markers, including lipid peroxidation and SOD activity (Fig. 8E, F, H-J). ATP levels in hyperuricemic mouse hearts were significantly lower but were restored by these treatments (Fig. 8K).

Echocardiography was used to assess cardiac function, with representative images shown in Fig. 8G. Hyperuricemic mice showed reduced LVEF and LVFS and increased LVIDs and LVESV. Treatment with allopurinol, ruxolitinib, or S3I-201 significantly improved LVEF and LVFS while reducing LVIDs and LVESV (Fig. 8L-O). Treadmill fatigue tests revealed impaired exercise capacity in hyperuricemic mice, with reduced running distance and time (Supplementary Fig. 2C, D). Although treatment improved both parameters, the differences were not statistically significant.

In conclusion, these findings demonstrate that inhibiting the JAK2/STAT3/HMGCS2 signaling pathway with ruxolitinib and S3I-201 alleviates mitochondrial dysfunction, oxidative stress, and cardiac dysfunction caused by hyperuricemia.

## Discussion

In this study, we investigated the mechanisms underlying uric acid-induced mitochondrial dysfunction and oxidative stress in cardiomyocytes. (1) We demonstrated that HMGCS2 plays critical roles in uric acid-induced mitochondrial fragmentation, functional disruption, increased oxidative stress, and reduced ATP production in cardiomyocytes. (2) Hyperuricemia was found to be associated with increased IL-6 expression, which may indicate activation of the JAK2/STAT3 signaling pathway. (3) ​ ​JAK2 phosphorylates STAT3 at the tyrosine 705 (Y705) residue, leading to its activation as a transcription factor, which in turn regulates the transcription of HMGCS2. (4) Furthermore, inhibiting the activation of JAK2 and STAT3 may modulates HMGCS2 expression, thereby alleviating uric acid-induced mitochondrial dysfunction and oxidative stress, and potentially improving cardiac dysfunction.

Increasing evidence suggests that hyperuricemia exacerbates cardiovascular diseases. Studies have shown that uric acid can induce monocyte migration into the myocardium, resulting in cardiac hypertrophy and inflammatory responses (Xu et al. 2021). It has also been demonstrated that uric acid impairs endothelial function by reducing nitric oxide synthesis and inhibiting endothelial cell proliferation, thereby promoting the development and progression of cardiovascular disease (Kang et al. 2005). However, the precise mechanisms by which uric acid damages cardiomyocytes have yet to be fully elucidated. As highly dynamic organelles within the cell, mitochondria continuously undergo cycles of fission and fusion. Mitochondrial dynamics is the key for mitochondria function and ensure the normal operation of cardiomyocytes. This study revealed that high uric acid levels induce an increase in the expression of the mitochondrial fission-related protein Fis1, along with a reduction in the expression levels of the mitochondrial fusion-related proteins MFN1 and MFN2 in cardiomyocytes. Mitochondrial staining further revealed that hyperuricemia disrupts the mitochondrial network, leading to mitochondrial fragmentation. An increase in mitochondrial fission can trigger mitochondrial autophagy (Shirihai et al. 2015). Research has indicated that hyperuricemia increases mitophagy in cardiomyocytes (Gao et al. 2021). Hyperuricemia induces increased mitophagy in cardiomyocytes, which may be attributed to the disruption of mitochondrial dynamics. Additionally, hyperuricemia significantly induced a decrease in mitochondrial membrane potential, an increase in oxidative stress levels, and a reduction in ATP production in AC16 cardiomyocytes and myocardial tissue from model mice.

To further investigate the mechanisms underlying these observations, RNA transcriptome analysis was conducted on mice cardiac tissue. The results revealed that the expression of the *HMGCS2* gene was significantly upregulated in hyperuricemic mice, showing a 5.12-fold increase compared with the control group. HMGCS2, expressed in mitochondria, is a rate-controlling enzyme in ketogenesis. Currently, several studies have reported the expression of HMGCS2 in cardiac tissue. In particular, studies have shown that HMGCS2 expression is elevated in diabetic cardiomyopathy, contributing to disease progression (Peng et al. 2023; Chen et al. 2022; Wang et al. 2024). Moreover, knocking out HMGCS2 in cardiomyocytes has been shown to significantly alleviate high glucose-induced oxidative stress and mitochondrial dysfunction (Chen et al. 2022). Similarly, Knockdown of HMGCS2 can alleviate the hyperglycemia-induced mitochondrial fission and mitochondrial dysfunction of glomerular endothelial cells (Shen et al. 2024). Given that hyperuricemia is the second most prevalent metabolic disorder after diabetes, the increased expression of HMGCS2 in hyperuricemic mice may indicate its potential role in hyperuricemia-induced oxidative stress and mitochondrial dysfunction, ultimately leading to cardiac dysfunction. Similarly, experimental results further confirmed that knocking out HMGCS2 in AC16 cardiomyocytes significantly reduces hyperuricemia-induced mitochondrial fission, restores mitochondrial membrane potential and ATP production, and decreases oxidative stress levels. These findings suggest that uric acid-mediated changes in HMGCS2 expression may contribute to hyperuricemia-induced cardiac dysfunction. However, the mechanisms by which uric acid regulates HMGCS2 expression remain unclear.

Epidemiological studies revealed a significant positive association between uric acid and inflammatory markers, with a progressive increase in the percentage of subjects exhibiting elevated levels of IL-6 and CRP across uric acid quintiles (Ruggiero et al. 2006). Moreover, uric acid levels was positively correlated with cardiac injury, which was observed in early-stage chronic kidney disease (CKD) rats with hyperuricemia (Bao et al. 2014). In our study, hyperuricemia significantly induced IL-6 mRNA expression in AC16 cardiomyocytes, and immunohistochemical analysis of myocardial tissue from hyperuricemic mice also revealed increased IL-6 levels. STAT3 is a transcription factor activated by various stimuli, such as IL-6 (Owen et al. 2019). The JAK2/STAT3 signaling pathway is a key regulator of various cellular functions, including survival, proliferation, and differentiation (Qin et al. 2018), and its persistent activation has been implicated in the progression of several diseases, such as interstitial lung diseases (Montero et al. 2021) and osteoarthritis (Chen et al. 2023). STAT3 and JAK2 are also involved in numerous cardiovascular diseases, including diabetic cardiomyopathy (Ji et al. 2024), atherosclerosis (Guo et al. 2024), pathological myocardial hypertrophy and fibrosis (Zhang et al. 2019), and lipopolysaccharide-induced myocardial injury (Fang and Guan 2024). Several studies have shown that the JAK2/STAT3 signaling pathway is involved in hyperuricemia-induced nephropathy (Ren et al. 2021a; Ren et al. 2021b) and that inhibiting this pathway can improve hyperuricemia-induced renal injury (Mehmood et al. 2022) and fibrosis (Sun et al. 2023). Our study revealed that hyperuricemia significantly increased the phosphorylation levels of JAK2 and STAT3 in AC16 cardiomyocytes and myocardial tissue from hyperuricemic mice, whereas the total protein levels of JAK2 and STAT3 remained unchanged.

Notably, this study found that uric acid induces IL-6 expression in cardiomyocytes without significantly affecting the expression of GP130 or IL-6R, suggesting that uric acid may activate the JAK2/STAT3 signaling pathway by increasing IL-6 levels. IL-6 can activate STAT3 through either the membrane-bound IL-6 receptor (mIL-6R, classical pathway) or the soluble IL-6 receptor (sIL-6R, trans-signaling pathway) (Wolf et al. 2014). At low concentrations, sIL-6R binds to IL-6 and exerts an antagonistic effect, while high concentrations of the IL-6/sIL-6R complex activate membrane gp130, leading to subsequent JAK/STAT3 phosphorylation (Jiang et al. 2021; Shi et al. 2020). Therefore, we introduced sIL-6R to demonstrate that hyperuricemia-induced STAT3 activation is associated with IL-6. We found that low concentrations of sIL-6R inhibited uric acid-induced STAT3 phosphorylation, whereas high concentrations of sIL-6R increased it. These findings suggest that the activation of STAT3 by uric acid may be partially mediated by IL-6. Furthermore, our research demonstrated that knocking out STAT3 in AC16 cardiomyocytes significantly reduced hyperuric acid-induced mitochondrial fission, restored the mitochondrial membrane potential and ATP production levels, and decreased oxidative stress. Similarly, treatment with the JAK inhibitor ruxolitinib also significantly lowered the phosphorylation levels of JAK2 and STAT3 induced by hyperuricemia, achieving effects comparable to those of STAT3 knockout. These findings highlight a close link between hyperuricemia, inflammation, mitochondrial dysfunction, and cardiomyocyte abnormalities, suggesting that the JAK2/STAT3 signaling pathway may play a central role in this relationship.

HMGCS2 is regulated by various transcription factors and posttranslational modifications (Kim et al. 2019; Li et al. 2021; Nadal et al. 2002). Silencing peroxisome proliferator-activated receptor alpha reduces HMGCS2 expression and alleviates myocardial injury in diabetic cardiomyopathy. Additionally, Wang et al. confirmed through dual-luciferase reporter assays that miR‐363‐5p regulates HMGCS2 expression and contributes to the progression of diabetic cardiomyopathy (Wang et al. 2020). However, the mechanisms underlying the upregulation of HMGCS2 in hyperuricemic cardiomyocytes remain unclear. STAT3, as a transcription factor, dimerizes upon phosphorylation and moves to the nucleus to regulate gene transcription (Levy and Lee 2002; Haghikia et al. 2014). Our results revealed that uric acid significantly induces the nuclear translocation of STAT3. Furthermore, knocking down STAT3 markedly inhibited the protein expression level of HMGCS2 induced by high uric acid, whereas overexpression of the STAT3 plasmid with sustained phosphorylation at residue 705 significantly upregulated HMGCS2 expression. Using the JASPAR database, we identified STAT3 binding sites in the promoter region of HMGCS2, and these findings were confirmed via dual-luciferase reporter assays. To our knowledge, we presented for the first time that STAT3 is a potential transcription factor for HMGCS2. Moreover, our findings demonstrated that both the knockdown of STAT3 and treatment with the JAK inhibitor ruxolitinib significantly reduced HMGCS2 expression induced by high uric acid. The overexpression of HMGCS2 in cardiomyocytes can reverse the effects of STAT3 knockdown and JAK inhibition on mitochondrial dysfunction, oxidative stress, and ATP generation abnormalities induced by high uric acid. These results suggest that in hyperuricemic cardiomyocytes, the JAK2/STAT3 signaling pathway may be activated by IL-6, leading to increased HMGCS2 expression, mitochondrial fission, functional impairment, and elevated oxidative stress, ultimately contributing to abnormal energy metabolism. Thus, intervening in the JAK2/STAT3 signaling pathway through either STAT3 knockout or JAK inhibition could potentially ameliorate uric acid-induced mitochondrial dysfunction in cardiomyocytes.

The animal experiment results in this study also partially corroborated the aforementioned effects. Treatment with the STAT3 inhibitor S3I-201 and the JAK inhibitor ruxolitinib significantly restored cardiac function abnormalities in hyperuricemic model mice. Additionally, these treatments ameliorated mitochondrial dysfunction, oxidative stress, and ATP levels in the heart tissue of hyperuricemic mice. Interestingly, the positive control group treated with allopurinol exhibited effects similar to those observed with S3I-201 and ruxolitinib, potentially due to the reduction in serum uric acid levels in hyperuricemic model mice. However, this study found that S3I-201 and ruxolitinib did not reduce serum uric acid levels in hyperuricemic mice. Nonetheless, they alleviated cardiac dysfunction in these mice, suggesting that their therapeutic effects may be attributed to the anti-inflammatory properties of S3I-201 and ruxolitinib. These agents inhibit the JAK2/STAT3 signaling pathway in myocardial tissue, thereby interfering with uric acid-induced HMGCS2 expression. However, some limitations should be mentioned. First, the absence of gene knockout models prevents the exclusion of alternative compensatory mechanisms or off-target effects, limiting the ability to establish a definitive causal relationship between the JAK2/STAT3/HMGCS2 pathway and cardiac dysfunction. Second, the use of AC16 cells, rather than primary cardiomyocytes, may not fully replicate the physiological responses of native cardiac tissue, potentially affecting the accuracy of the findings. Third, Long-term animal studies are required to evaluate the safety and efficacy of ruxolitinib and S3I-201, as well as to assess their sustained therapeutic benefits and potential adverse effects. Therefore, future studies using HMGCS2 knockout mice to investigate the mechanisms of hyperuricemia, along with long-term animal studies to evaluate the effects of ruxolitinib and S3I-201 on cardiac function in hyperuricemic mice, may enhance the current findings and yield more insightful results.

## Conclusions

In summary, we observed that elevated uric acid levels may increase IL-6 expression in cardiomyocytes, which in turn could activate the JAK2/STAT3 signaling pathway and promote HMGCS2 transcription. Elevated HMGCS2 levels may lead to increased mitochondrial fission and functional abnormalities, contributing to oxidative stress and ATP production deficits in cardiomyocytes, ultimately potentially causing heart dysfunction. Intervention targeting the phosphorylation of JAK2 and STAT3 may inhibit the JAK2/STAT3/HMGCS2 signaling pathway, thereby alleviating hyperuricemia-induced mitochondrial dysfunction, oxidative stress, and energy metabolism disorders, and potentially improving heart dysfunction.

## Supplementary Information

Supplementary Material 1: Supplementary Figure 1. Uric acid stimulation induced the nuclear translocation of STAT3. A. Images display, respectively, FITC-conjugated anti-T-STAT3, DAPI, and Mergeantibodies. B-C. Representative Western blot imageand the corresponding statistical analysisof T-STAT3 in nuclear extracts prepared from mouse heart tissues. The data represent the means ± S.E.M.s. *p<0.05 vs the control group. #p<0.05 vs the hyperuricemia group. Supplementary Figure 2. Serum uric acid levels and exercise capacity in a hyperuricemic mouse model. A-B. Serum uric acid levels were assessed during the first weekand the sixth weekfollowing the induction of hyperuricemia. C‒D. The treadmill fatigue test, which was conducted in the sixth week posthyperuricemia induction, was used to evaluate running distanceand running time. The data are presented as the means ± S.E.M.s. *p<0.05 compared with the control group. #p<0.05 compared with the hyperuricemia group.

## Data Availability

No datasets were generated or analysed during the current study.
