# Supplementary figures and images for: JAK2/STAT3/HMGCS2 signaling aggravates mitochondrial dysfunction and oxidative stress in hyperuricemia-induced cardiac dysfunction
Source: Mol Med. 2025 May 13;31:184. doi: 10.1186/s10020-025-01246-x (PMC12070620; doi:10.1186/s10020-025-01246-x)

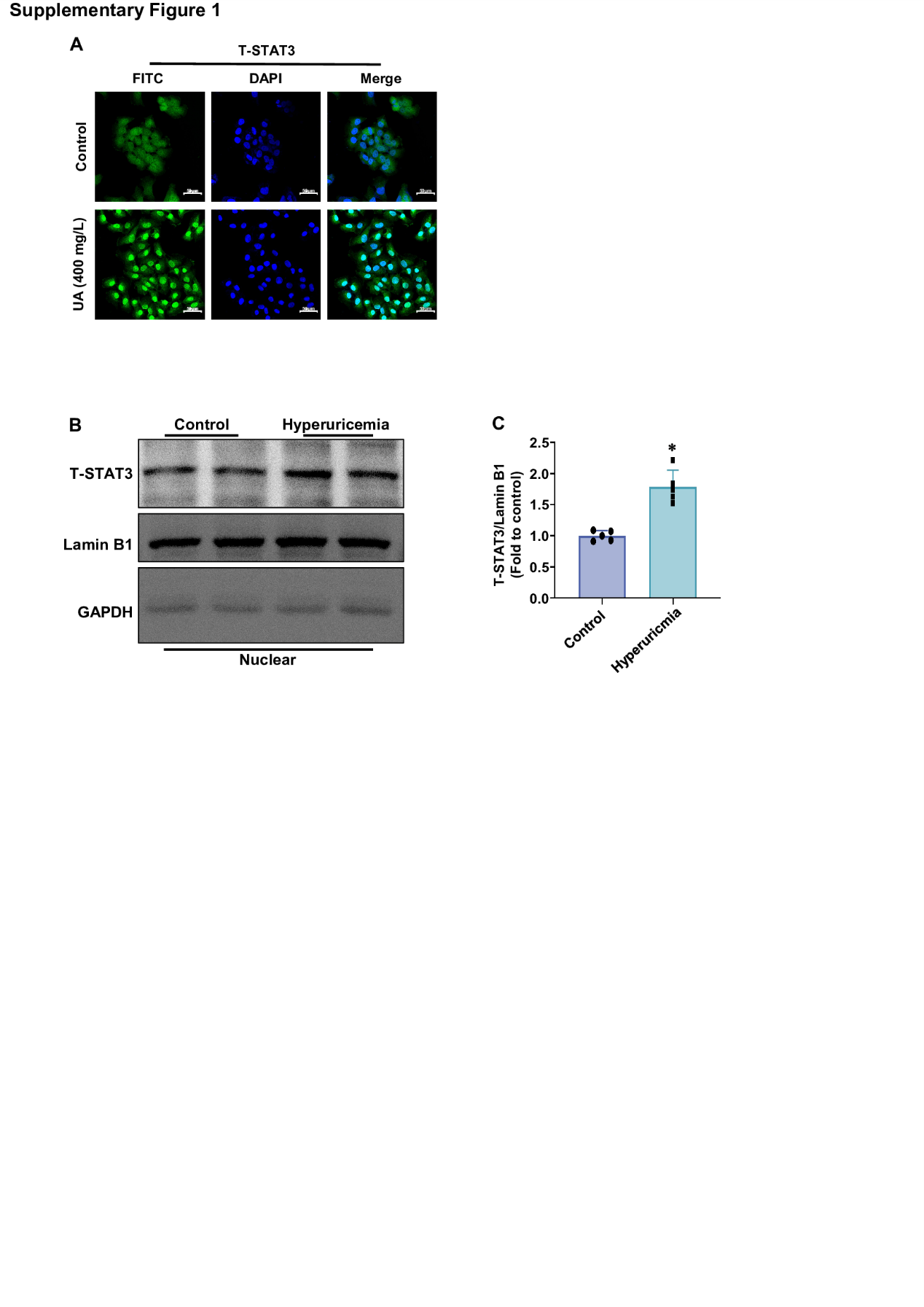


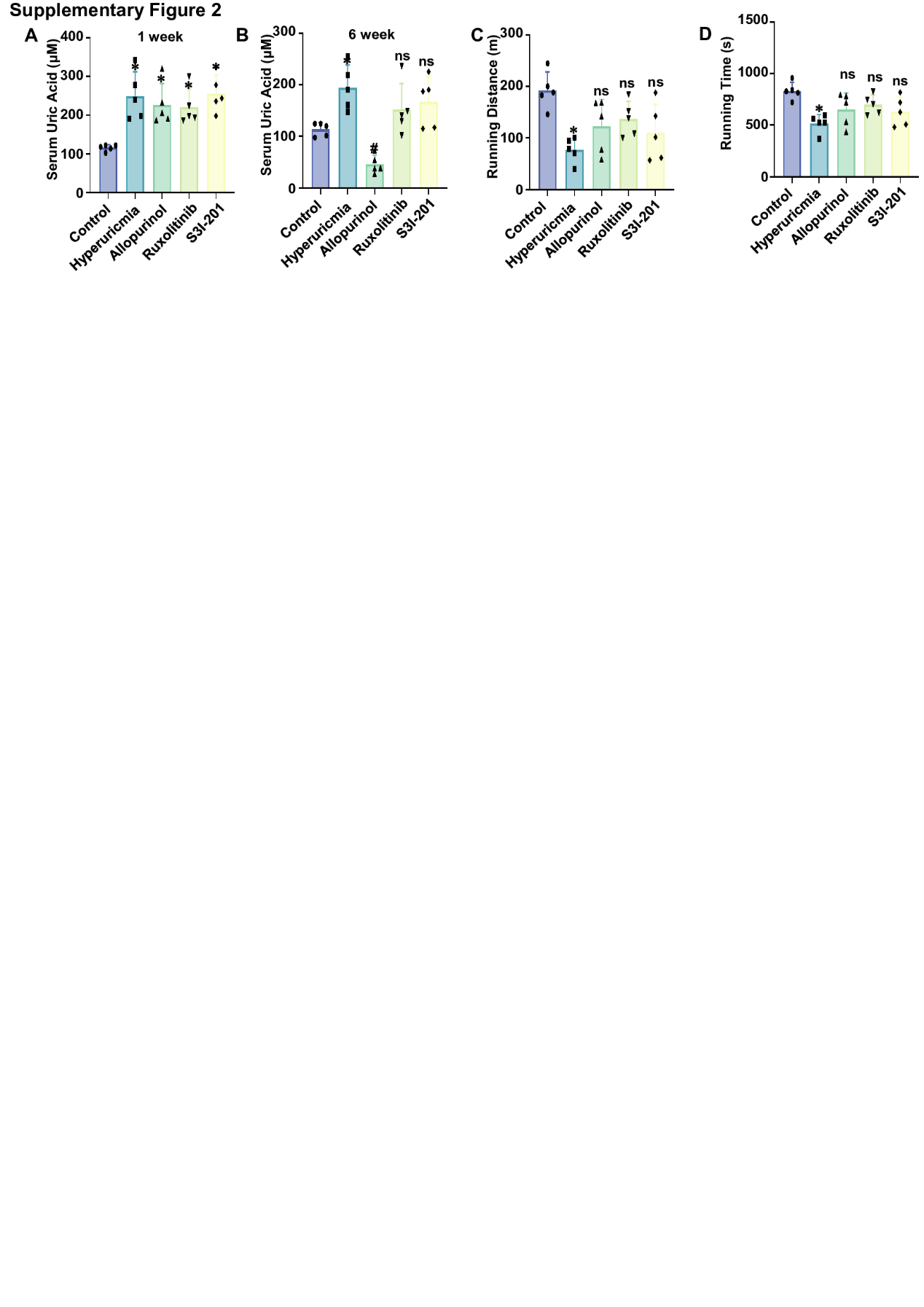

Supplement: Supplementary file 1 — Supplementary Material 1: Supplementary Figure 1. Uric acid stimulation induced the nuclear translocation of STAT3. A. Images display, respectively, FITC-conjugated anti-T-STAT3, DAPI, and Mergeantibodies. B-C. Representative Western blot imageand the corresponding statistical analysisof T-STAT3 in nuclear extracts prepared from mouse heart tissues. The data represent the means ± S.E.M.s. *p<0.05 vs the control group. #p<0.05 vs the hyperuricemia group. Supplementary Figure 2. Serum uric acid levels and exercise capacity in a hyperuricemic mouse model. A-B. Serum uric acid levels were assessed during the first weekand the sixth weekfollowing the induction of hyperuricemia. C‒D. The treadmill fatigue test, which was conducted in the sixth week posthyperuricemia induction, was used to evaluate running distanceand running time. The data are presented as the means ± S.E.M.s. *p<0.05 compared with the control group. #p<0.05 compared with the hyperuricemia group. [file 10020_2025_1246_MOESM1_ESM.docx]
